# Socioeconomic inequalities in use and non-use of dental services in Poland

**DOI:** 10.1007/s00038-020-01379-2

**Published:** 2020-05-09

**Authors:** Dorota Elżbieta Piotrowska, Dorota Jankowska, Dorota Huzarska, Andrzej Stanisław Szpak, Bartosz Pędziński

**Affiliations:** 1 Department of Public Health, Medical University of Bialystok, ul. Szpitalna 37, 15-295 Białystok, Poland; 2 Department of Statistics and Medical Informatics, Medical University of Bialystok, ul.Szpitalna 37, Białystok, 15-295 Poland; 3 Institute of Rural Health, ul.Jaczewskiego 2, Lublin, 20-090 Poland; 4 Lomza Medical Center Ltd., ul.Ks.Kardynała Wyszyńskiego 9, Lomza, 18-400 Poland

**Keywords:** Health disparities, Socioeconomic factors, Dental public health, Inequalities, Oral health

## Abstract

**Objectives:**

To assess the impact of classical socioeconomic factors on the use and non-use of dental services on a representative sample of Polish population.

**Methods:**

The study was based on face-to-face surveys conducted by GUS (Statistics Poland) on 13,376 respondents in 2010 and 12,532 individuals in 2013.

**Results:**

The percentage of people using dental services in the highest income group was approximately twice as high as that in the lowest one (Q1: 7.0% vs. Q5: 16.4%), with the same being true for education (the lowest education group: 8.3% vs. the highest education group: 18.0%), and place of residence (inhabitants of rural areas: 9.2% vs. inhabitants of largest cities: 15.9%) in 2013. The analysis has shown the disparities in not using dental services when in need to be less clear-cut.

**Conclusions:**

The conducted research, based on two independent periods, a representative population sample, univariate analysis and the multivariate regression model has revealed pronounced social inequalities in dental care use. It is a challenge to determine the factors which contribute most to health inequalities and the interventions which are most effective in reducing them.

## Introduction

Despite intensive efforts aimed to reduce health inequalities and implement universal health coverage, considerable health disparities are still clearly visible at national and international levels (WHO 2015). As numerous studies have shown, there is a dependency between socioeconomic status and general and oral health (Costa et al. 2012; Watt et al. 2015; Krzyżak et al. 2015). However, reports on relations between SES and use of dental services and the non-use while needed are scarce.

Dental care disparities might arise from a range of factors, one of them being the above-mentioned socioeconomic status. In comparison to people with low SES, high-SES individuals are characterised by better dental health behaviours, such as a higher frequency of teeth cleaning with greater care and with the use of additional hygiene products (Watt and Sheinham 1998; Jerkovic et al. 2009; Park et al. 2016). Apart from that, they display better nutritional behaviours and there are fewer smokers among them, which also reduces the risk of poor oral health (Mobley et al. 2009; Charkiewicz et al. 2018; Murakami et al. 2018). Moreover, such people have their dental check-ups and other health services more often, which is a feature attributable also to level of education (Palencia et al. 2014).

The inequalities result also from environmental factors. Inhabitants of rural areas face a worse availability of dental practices than residents of urban areas (Piotrowska et al. 2018; Emami et al. 2014, Brock Martin et al. 2012). Low-income people cannot afford the same level of medical care as more affluent ones can (Piotrowska et al. 2016). This is particularly manifest in dental care, where a large portion of services are excluded from the public financing mechanism. In nearly all OECD countries, disparities in the use of dental services determined by income and economic status are notably greater than those concerning the use of primary and hospital health care services (van Doorslaer and Masseria 2004; Devaux and de Looper 2012). According to the European Union Statistics on Income and Living Conditions (EU-SILC) 2014 data, the share of the population with unmet needs is greater for dental care (7.6%) than for medical care (6.7%). Although 95% of Polish population is covered by public health insurance and entitled to state-funded health care services, merely 26.8% of residents of Poland used such services within the public health insurance system and 74.2% of the population paid for them out of pocket in 2016 (GUS 2017). The reason for that is the cost of a number of dental services not being covered by NFZ (National Health Fund), e.g. endodontic treatment of posterior teeth in adults, or the waiting time being too long, or the quality of the services being so poor that patients decide to use them against payment.

The effect of the situation is disparities in oral health. Less affluent people are characterised by an increased incidence of dental caries and a higher number of missing teeth (Brennan et al. 2001). Individuals who pursued their education for more than 12 years typically experience a smaller extent of gingival bleeding, a lower loss of periodontal attachment, and fewer missing tooth surfaces than those whose schooling lasted for a shorter period (Sabbah et al. 2009).

The purpose of this study was to identify the influence of socioeconomic factors on using dental services and on not using them despite such a need based on a representative sample of Polish population. The analysis relied on classical SES factors, such as income, education, place of residence, and source of income.

## Methods

The data were derived from the study titled “Health care in households” carried out by GUS (Statistics Poland). It covered a representative sample of 4658 households of 13,376 members in total in 2010 and 4584 households of 12,532 members in total in 2013. It was conducted by means of a face-to-face survey as part of “Household budget survey”. The research tool comprised two questionnaires: a common one for a household and an individual one for its respective members.

The survey was carried out with a representative method, which enables generalisation, characterised by a certain precision, of the findings with respect to all households in Poland. The scheme applied was stratified two-stage sampling with various probabilities of selection at stage 1. Stage 1 sampling units were area survey points and stage 2 sampling units were dwellings. As some households do not participate in the study (they refuse to take part in the survey), the adopted method consisted in substituting the households selected by sampling yet choosing not to respond. The substitution was based on sequential sampling. In 2010, 33.9% of the examined household structure was composed of dwellings from the basic sample and the substituted sample constituted 66.1% of the selected households, whereas in 2013 it was 44.3% and 55.7%, respectively. The 2010 module survey “Health care in households” covered 98.7% of the households which took part in “Household budget survey”, while the 2013 result was 97.6%. A detailed description of the sampling scheme, the research method, the instruments used, and the methodology applied can be found in methodological studies by GUS (GUS 2010, 2013). The methods employed in the research enabled a comparison of the obtained results with the outcome of earlier module surveys of health care in households conducted by GUS and at the same time they allowed the use of its findings in a system of health accounts (OECD methodology: SHA 1.0 and SHA 2011) (OECD 2011; GUS 2017).

In 2010, the analysed group was composed of 13,376 people, including 6365 males and 7011 females. The median of the respondents’ age was 38 (min. 0, max. 102). In 2013, the sample consisted of 12,532 individuals, including 6012 males and 6520 females, whose median age was 41 (min. 0, max. 98). The information regarding subjects aged under 15 was provided by their parents or guardians.

The respondents were asked the following questions: Did you use dental services in the last quarter of 2010? (the same question for 2013)- i.e. use of dental care; Did you have a situation in 2010 where you did not use dental services despite being in need of them?(the same question for 2013), i.e. non-use of dental care. There were yes/no answer variants. From among the potential reasons for resignation despite a health need, only the answer variants selected by more than 10 respondents were analysed (long waiting time, lack of time, lack of money, fear of dental visit, I expected the problem to go away, other).

The dependency of using dental services and resigning from dental care despite such a need on income, source of income, education, and place of residence were examined. Household’s available income was defined by GUS as “the sum of the current incomes of the household from all sources reduced by advances towards personal income tax deducted by the employer on behalf of the tax-payer, by taxes paid from income from property, by taxes paid by self-employed persons, including freelance professionals and persons using private farm in agriculture and by social security and health insurance premiums.” (GUS 2013). It was analysed in PLN (Polish Zloty) by quintile groups in 2010: I: ≤ 644.88; II: 645.00–898.57; III: 899.00–1182.61; IV: 1182.65–1600.64; V: ≥ 1601.67; and in 2013: I: ≤ 722.50; II: 723.31–1033.75; III: 1033.93–1350.00; IV: 1350.08–1850.00; V: ≥ 1850.20. The main source of income, divided by GUS into nine classes, was arranged into six classes: cl. 1: wages and salaries; cl. 2: farm income; cl. 3: self-employment; cl. 4: annuities and pensions; cl. 5: social benefits; cl. 6: other income and being a dependant. Education was classified in accordance with the International Standard Classification of Education (ISCED 2011): low education: level 0–2, medium education: level 3–4, high education: level 5–8. People under 19 were excluded from the analysis of level of education since its impact on dental care use is very limited in their case because they are still pursuing their education. Place of residence was classified in accordance with the GUS division: 500 thousand inhabitants and more; 200–500 thousand; 100–200 thousand; 20–100 thousand; 20 thousand and fewer; village.

To assess the statistical significance of the relation between categorical characteristics, Pearson’s Chi-squared test was applied. Subsequently, a univariate logistic regression analysis and a multivariate logistic regression analysis were performed. The latter was carried out at two stages, with the first one including the socioeconomic factors (income, education, place of residence, source of income) and interactions between gender and these factors (gender was treated as an adjustment variable) as well as an interaction between income and education. Due to the fact that the model included too many variables (most of which had no statistically significant influence on the use of and resignation from dental services), the second step was taken. It preserved the same SES factors, adjustment variables, i.e. gender and age (the impact of which on inequalities in non-dental health), as well as income–education and income–gender interactions. This model, as the final one, is presented in this study. The normality of the distribution of the analysed income was assessed with the Shapiro–Wilk test. Since the distribution was not normal, income was compared in the examined groups by means of the Mann–Whitney U test. The results at the level of *p* ≤ 0.05 were considered statistically significant. STATA/IC 12.1 by StataCorp LP. and the Statistica 12.5 package by StatSoft were used for the calculations.

## Results

**Fig. 1:**
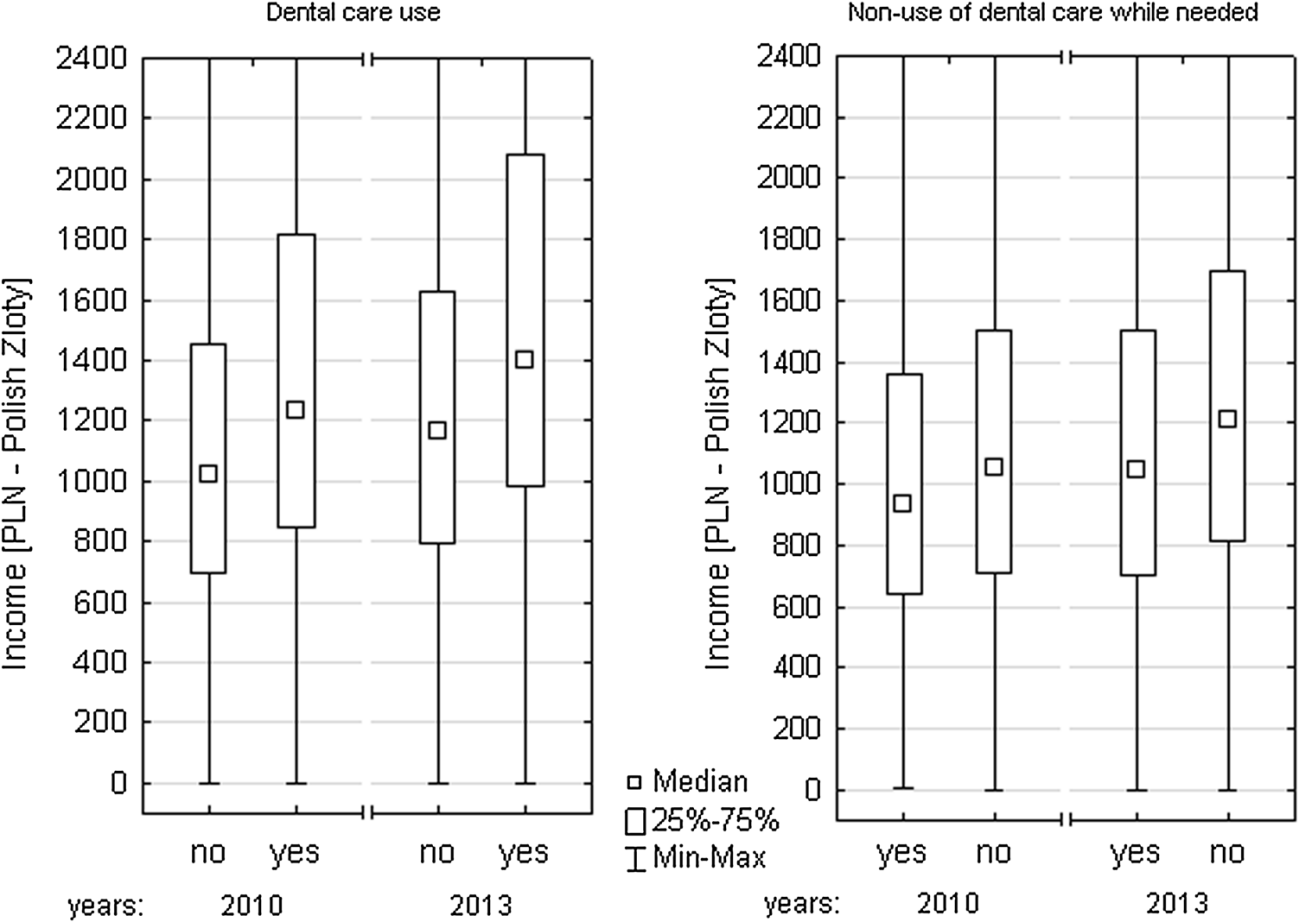
Income associated with use and non-use of dental care while needed (Poland 2010 and 2013)

Statistically significant differences between those who did and did not use dental services were disclosed in terms of level of income in both analysed years (*p* < 0.001). In 2010, the median of the income generated by the people who did not use dental care was PLN 1019.50 and that of the individuals who did amounted to PLN 1235.24. Similarly, the 2013 income median in the group of non-users was PLN 1164.20, and in the group of users—PLN 1400.00. The 2010 median of income earned by those who did not use such services despite a health need was PLN 935.50, while in the case of those who did not experience such a problem it amounted to PLN 1053.50. In 2013, it was PLN 1044.87 and PLN 1207.50, respectively (*p* < 0.001) (Fig. 1).

A statistically significant dependency of the use of dental care on all socioeconomic factors included in the research was ascertained in both analysed years. The fact of resigning from dental treatment despite such a need also proved important dependence on income level source of income, education, and place of residence (in 2010, *p* = 0.02; in 2013, at the limit of statistical significance: *p* = 0.05) in both analysed years (Tables 1, 2).

**Table 1:** Socioeconomic factors associated with use of dental care (Poland 2010 and 2013)

	2010		2013	
	Use of dental care		Use of dental care	
	Persons	%	Persons	%
*Income-classes*				
Lowest	182	6.8	176	7.0
II	236	8.9	206	8.3
III	275	10.3	248	9.9
IV	294	11.0	286	11.5
Highest	461	17.3	410	16.4
	*p* < 0.0001		*p* < 0.0001	
*Education*				
Low	260	8.7	210	8.3
Medium	681	10.2	616	9.7
High	311	18.7	333	18.0
	*p* < 0.0001		*p* < 0.0001	
*Place of residence*				
500 Thousand and more	224	16.6	208	15.9
200–500 Thousand	150	13.3	146	14.1
100–200 Thousand	97	11.5	78	9.3
20–100 Thousand	239	11.3	181	9.7
20 Thousand and fewer	136	9.1	156	11.5
Village	609	9.4	562	9.2
	*p* < 0.0001		*p* < 0.0001	
*Source of income*				
Wages and salaries	502	12.4	486	12.3
Farm income	63	9.6	59	9.6
Self-employment	66	13.6	50	11.2
Annuities and pensions	251	7.7	259	8.3
Social benefits	51	9.2	58	11.7
Other sources	526	12.3	419	10.7
	*p* < 0.0001		*p* < 0.0001

**Table 2:** Socioeconomic factors associated with non-use of dental care while needed (Poland 2010 and 2013)

	2010		2013	
	Resignation despite such a need		Resignation despite such a need	
	Persons	%	Persons	%
*Income-classes*				
Lowest	269	10.6	144	6.3
II	215	8.5	116	5.1
III	223	8.7	91	4.0
IV	155	6.1	99	4.3
Highest	159	6.2	78	3.3
	*p* < 0.0001		*p* < 0.0001	
*Education*				
Low	222	7.7	97	4.2
Medium	635	10.0	352	6.0
High	128	8.0	65	3.8
	*p* = 0.0005		*p* < 0.0001	
*Place of residence*				
500 Thousand and more	108	8.5	65	5.4
200–500 Thousand	88	7.9	47	4.9
100–200 Thousand	84	11.2	47	6.3
20–100 Thousand	155	7.8	75	4.5
20 Thousand and fewer	97	6.8	46	3.6
Village	490	7.9	248	4.4
	*p* = 0.02		*p* = 0.05	
*Source of income*				
Wages and salaries	440	11.2	210	5.8
Farm income	77	12.0	37	6.3
Self-employment	40	8.7	13	3.2
Annuities and pensions	184	5.8	121	4.1
Social benefits	69	12.8	37	7.9
Other sources	212	5.3	110	3.2
	*p* < 0.0001		*p* < 0.0001	

The analysis of the use of dental services in individual quintile groups showed that people with the highest income used such services over twice as often as those with the lowest income in both years. In 2010, it was 17.3% of the wealthiest and 6.8% of the poorest, and in 2013—16.4% and 7.0%, respectively. People from the lowest quintile group resigned most frequently (10.6% in 2010 and 6.3% in 2013), while those from the highest income group resigned least frequently (6.2% in 2010 and only 3.3% in 2013). As regards education, the difference in the frequency of using dental services between the extreme groups was similar to that for income. In 2010, 18.7% of the respondents from the highest education group and only 8.7% of those from the lowest education group used dental services. In 2013, it was 18.0% and 8.3%, respectively. Contrary to expectations, the highest percentage of those who resigned from dental services was revealed in the medium rather than the lowest education group. Residents of the largest cities (circa 16%) used dental services most frequently, while people living in villages did it least often (circa 9%) in both years under examination. In 2010, inhabitants of medium-sized cities (11.2% of the subjects in 2010, 6.3%—in 2013) resigned from the services most frequently, whereas in 2013 the dependency of resignation from such services on place of residence was at the limit of statistical significance (*p* = 0.05). In both analysed years, the lowest percentage of dental care users was in the group of annuitants and pensioners: 7.7% in 2010 and 8.3% in 2013. The highest level of use was observed in the group of self-employed (13.6% in 2010) and employees (12.4% in 2010 and 12.3% in 2013). Social welfare beneficiaries (12.8% in 2010 and 7.9% in 2013) and farmers (12.0 in 2010 and 6.3 in 2013) resigned from dental care most often.

**Fig. 2:**
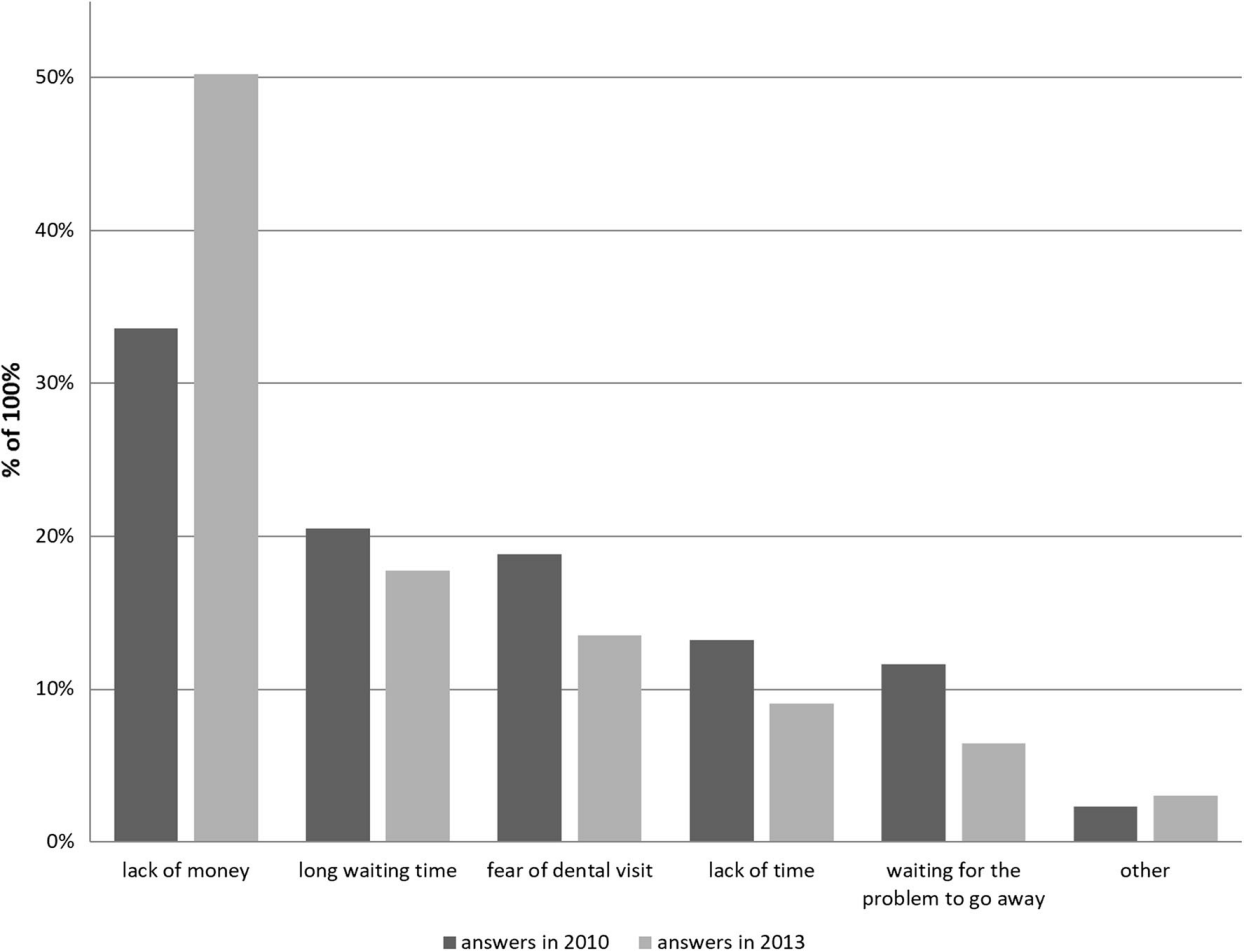
Reasons for non-use of dental care while needed (Poland 2010 and 2013)

The most common reason for not using dental services despite such a need was lack of money (33.6% in 2010 and 50.2% in 2013). Long waiting time accounted for 20.5% in 2010 and 17.7% in 2013; fear of dental visit—18.8% and 13.5%; lack of time—13.2% and 9.1%; waiting for the problem to get better on its own—11.6% and 6.5%; other—2.3 and 3.0%, respectively (Fig. 2).

Additionally, the influence of socioeconomic factors on use and resignation was examined with univariate and multivariate logistic regression models (Tables 3, 4). The univariate analysis showed that, in both years under examination, the odds of using dental services grew for females, for those living in bigger cities, along with the increasing level of income and education, and they dropped along with age and for annuitants and pensioners as compared to waged and salaried people. In 2010, dental services were used less frequently by individuals living on social benefits, and in 2013—by those with other sources of income. As regards non-use, in both analysed years, the univariate analysis showed that the odds of resignation grew slightly along with age and dropped along with the increasing income and in the group of people living on annuities and pensions and other sources with respect to the group of individuals living on wages and salaries. In 2013, the odds of resignation grew along with the population of the respondents’ places of residence, and dropped for the self-employed in relation to the waged and salaried.

**Table 3:** Univariate and multivariate logistic regression models of socioeconomic factors associated with use of dental care (Poland 2010 and 2013)

	2010				2013			
	Univariate		Multivariate		Univariate		Multivariate	
	OR (95% CI)	*p*	OR (95% CI)	*p*	OR (95% CI)	*p*	OR (95% CI)	*p*
Gender (female vs. male)	1.431 (1.281–1.599)	< **0.001**	1.556 (1.061–2.281)	**0.024**	1.527 (1.359–1.715)	< **0.001**	2.186 (1.484–3.220)	< **0.001**
Age (in years)	0.991 (0.988–0.993)	< **0.001**	0.981 (0.976–0.987)	< **0.001**	0.994 (0.992–0.997)	< **0.001**	0.989 (0.983–0.994	< **0.001**
Income (5 quintile groups^a^)	1.277 (1.227–1.329)	< **0.001**	1.547 (1.260–1.900)	< **0.001**	1.266 (1.215–1.320)	< **0.001**	1.316 (1.072–1.614)	**0.009**
Level of education (3 ordinal categories^b^)	2.138 (1.915–2.386)	< **0.001**	2.339 (1.631–3.353)	< **0.001**	2.071 (1.852–2.317)	< **0.001**	1.584 (1.118–2.246)	**0.010**
Place of residence (3 ordinal categories^c^)	1.304 (1.221–1.393)	< **0.001**	1.111 (1.021–1.207)	**0.015**	1.266 (1.181–1.356)	< **0.001**	1.077 (0.989–1.173)	0.089
*Source of income*^d^								
Farm income	0.774 (0.587–1.020)	0.069	1.231 (0.913–1.660)	0.172	0.757 (0.569–1.006)	0.055	1.170 (0.860–1.591)	0.316
Self-employment	1.149 (0.872–1.514)	0.324	1.256 (0.947–1.667)	0.115	0.893 (0.655–1.216)	0.472	0.954 (0.696–1.308)	0.772
Annuities and pensions	0.607 (0.518–0.712)	< **0.001**	1.170 (0.935–1.462)	0.169	0.640 (0.546–0.751)	< **0.001**	1.014 (0.811–1.267)	0.905
Social benefits	0.735 (0.543–0.995)	**0.047**	0.867 (0.586–1.281)	0.471	0.945 (0.707–1.263)	0.704	1.227 (0.856–1.758)	0.265
Other sources	1.022 (0.897–1.165)	0.742	1.183 (0.967–1.445)	0.099	0.854 (0.743–0.982)	**0.026**	0.982 (0.794–1.216)	0.870
Income_level of education			0.901 (0.232–0.987)	**0.023**			0.997 (0.912–1.089)	0.939
Gender_income			1.000 (0.905–1.105)	0.996			0.910 (0.822–1.007)	0.068

*p* < 0.05 is considered statistically significant (in bold)

^a^In 2013: I: ≤ 644.88; II: 645.00–898.57; III: 899.00–1182.61; IV: 1182.65–1600.64; V: ≥ 1601.67. In 2013: I: ≤ 722.50; II: 723.31–1033.75; III: 1033.93–1350.00; IV: 1350.08–1850.00; V: ≥ 1850.20

^b^Low: primary and lower secondary; Medium: upper secondary and post-secondary non-tertiary; High: short-cycle tertiary, bachelor, master and doctoral

^c^Place of residence: up to 20 thousand inhabitants; 20–200 thousand inhabitants; 200 thousand inhabitants and more

^d^Reference category: wages and salaries

**Table 4:** Univariate and multivariate logistic regression models of socioeconomic factors associated with non-use of dental care while needed (Poland 2010 and 2013)

	2010				2013			
	Univariate		Multivariate		Univariate		Multivariate	
	OR (95% CI)	*p*	OR (95% CI)	*p*	OR (95% CI)	*p*	OR (95% CI)	*p*
Gender (female vs. male)	0.998 (0.878;1.134)	0.980	0.995 (0.730–1.356)	0.974	1.046 (0.878–1.246)	0.614	1.194 (0.790–1.806)	0.400
Age (in years)	1.004 (1.001;1.007)	**0.004**	0.995 (0.989–1.001)	0.079	1.008 (1.004;1.012)	< **0.001**	1.001 (0.993–1.009)	0.861
Income (5 quintile groups^a^)	0.858 (0.819;0.898)	< **0.001**	0.642 (0.535–0.771)	< **0.001**	0.855 (0.803;0.911)	< **0.001**	0.853 (0.662–1.099)	0.218
Level of education (3 ordinal categories^b^)	0.943 (0.841;1.057)	0.311	0.641 (0.481–0.854**)**	**0.002**	0.875 (0.750;1.019)	0.087	0.928 (0.628–1.369)	0.706
Place of residence (3 ordinal categories^c^)	1.052 (0.970;1.139)	0.221	1.186 (1.079–1.303)	< **0.001**	1.127 (1.012;1.255)	**0.029**	1.305 (1.154–1.475)	< **0.001**
*Source of income*^d^								
Farm income	1.080 (0.835;1.398)	0.557	1.043 (0.795–1.368)	0.763	1.098 (0.765;1.575)	0.613	1.041 (0.711–1.524)	0.837
Self-employment	0.754 (0.537;1.059)	0.103	0.827 (0.587–1.165)	0.277	0.535 (0.302;0.945)	**0.031**	0.566 (0.319–1.004)	0.051
Annuities and pensions	0.488 (0.408;0.583)	< **0.001**	0.554 (0.435–0.706**)**	< **0.001**	0.691 (0.550;0.869)	**0.002**	0.641 (0.470–0.873)	**0.005**
Social benefits	1.158 (0.883;1.519)	0.288	1.160 (0.860–1.566)	0.331	1.398 (0.972;2.011)	0.071	1.287 (0.870–1.906)	0.208
Other sources	0.442 (0.373;0.524)	< **0.001**	0.729 (0.586–0.908)	**0.005**	0.535 (0.423;0.677)	< **0.001**	0.885 (0.667–1.176)	0.399
Income_level of education			1.115 (1.026–1.212)	**0.011**			0.981 (0.874–1.101)	0.745
Gender_income			1.011 (0.917–1.114)	0.824			0.959 (0.840–1.093)	0.525

*p* < 0.05 is considered statistically significant (in bold)

^a^In 2013: I: ≤ 644.88; II: 645.00–898.57; III: 899.00–1182.61; IV: 1182.65–1600.64; V: ≥ 1601.67. In 2013: I: ≤ 722.50; II:723.31–1033.75; III: 1033.93–1350.00; IV: 1350.08–1850.00; V: ≥ 1850.20

^b^Low: primary and lower secondary; medium: upper secondary and post-secondary non-tertiary; high: short-cycle tertiary, bachelor, master and doctoral

^c^Place of residence: up to 20 thousand inhabitants; 20–200 thousand inhabitants; 200 thousand inhabitants and more

^d^Reference category: wages and salaries

The multivariate analysis confirmed that, in both examined years, the odds of using dental care grew for females, along with the increasing level of education and income (with other factors established in the model). The odds slightly decrease, in turn, along with the respondents’ age. In 2010, the odds of use were enhanced by living in larger cities and reduced by the income–education interaction. The multivariate approach to the non-use of dental care showed a significant influence of merely two of the analysed variables in both years under examination. The odds of non-use of dental care grew along with the increasing population of the respondent’s place of residence, and dropped for those whose source of income was annuities and pensions in comparison to the respondents living on wages and salaries. It was also observed in 2010 that the increase in income (OR = 0.64) and education (OR = 0.64) and having other sources of income considerably reduced the odds of resigning from dental care. Apart from the independent impact of income and education on resignation, the interaction of these factors also plays a major role. The relation between the non-use of dental care and income is moderated by education (OR = 1.11 for the interaction shows that the decrease in the odds of resigning from dental care along with the increasing income and education is not as sharp as it could seem if only the two independent socioeconomic factors were taken into consideration in the model.

## Discussions

The analysis based on Pearson’s Chi-squared test, univariate and multivariate logistic regression models showed that, in both analysed years, there were strong and consistent dependencies between the use of dental care and level of income and level of education. As regards the non-use of dental care, this dependency was not that clear in both examined years and with all employed statistical approaches taken into consideration.

The basic limitation of this study is the fact that the analysis of the non-use of dental care while needed was based on a subjective determination whether there was such a need without a clinical examination of a patient’s health. It can be expected that an objective percentage of the patients who should use dental care was higher since patients often show up only when they have experienced late symptoms (e.g. gingival bleeding or acute pain) and the intervention should have been sought much earlier. Another limitation is the distant period of study, but these were the most recent data available from GUS. Although the current dependencies between SES and the use of dental services might be different, this does not undermine the value of the observations made over the analysed years. A major shortcoming of the survey conducted by GUS is the question about using dental services by respondents over the last quarter of a year as it seems to disregard the fact that the use of such services is seasonal due to both patients’ behaviours and reduced supply of medical services towards the end of a year as a result of the limitations imposed on services financed by NFZ.

The strengths of the study include the use of a large representative sample of Polish population, the adopted methodology of a simultaneous comparison of the general use and non-use of dental services, and the inclusion of two independent periods (years) in the analysis. Moreover, it employs both univariate assessment and the multivariate logistic regression analysis.

The results of our research confirm the global trends which point to the fact that individuals with higher income use dental services more often. This dependency was proved in the univariate and multivariate analyses. Minor disproportions in this respect are noticeable in Finland, where 61% of people with the lowest income and 72% with the highest income use dental care (Raittio et al. 2015), whereas the greatest disproportions were observed in Canada, where people with the highest income used dental care three times more often than those with the lowest income (Bhatti et al. 2007). For the analysed population of Poland, it was proved that the differences in the use of dental services in the highest income group were twice as high as in the lowest one, while the differences in non-use despite such a need were less visible. This is confirmed also by the median of income in the studied groups. Such a dependency could attest to the fact that people with the lowest income are more willing to cover the cost of curative dental services than preventive ones. The inequalities resulting from economic status are confirmed by the respondents asked about the reason for their non-use of dental services. In the 2013 GUS survey, a half of the Polish population pointed to shortage of funds as the reason for their resignation from dental care. In a European survey, “too expensive” is the reason for unmet needs for dental examination or treatment in two thirds of all EU citizens (Eurostat 2017a).

In the case of education, the differences in the use of dental services between the extreme groups were considerable, which coincides with the results noticeable in other European countries (European Commission 2010). In our study, the highest percentage of non-users despite a health need was revealed in the medium education group, which is an exception to the trend noticed in the EU-SILC research (Eurostat 2017b). Moreover, level of education did not prove to be a significant factor in deciding not to use dental services in the multivariate logistic regression model. Goulart and Vettore (2016) showed that in the case of people with at least 11 years of education severe tooth loss was 0.9%, whereas for those with less than 4 years of schooling it was 9%. Also, Chaves and Vieira-da-Silva (2008) found that the lowest DMFT (Decayed Missing Filled Teeth) index was in the highest education group. The analyses of the univariate and multivariate logistic regression analyses confirmed that there was a dependency between use and education in both years under examination. As regards resignation, this dependency was noticeable only in the multivariate regression model in 2010.

The most frequent users of dental services are inhabitants of the largest cities, while the least frequent ones are people living in villages. Yet the analysis of the non-use of dental care when in need showed that the problem usually concerned inhabitants of medium-sized cities. The results should be interpreted with caution due to the fact that the dependency of the resignation from a visit on the place of residence was at the limit of statistical significance. The situation of inhabitants of villages (who do not necessarily need to be farmers) requires a special analysis as in our research such persons were characterised by the least frequent use of services in general, yet they did not resign from the services when in need more often than inhabitants of cities. Some studies indicate that inequalities in access to health care between cities and villages have been more typical of underdeveloped rather than developed countries (Watt et al. 2015). Currently, there are limited data in this regard in the population of adults in Poland, but certain publications point to considerable oral health negligence in the case of children (Brock Martin et al. 2012; Czapiński and Panek 2015). The univariate logistic regression analysis showed that the odds of using dental care grew along with the increasing population of the respondents’ place of residence in 2010 and 2013, while the multivariate analysis revealed this dependency in 2010. As regards non-use, this dependency was noticeable in the univariate model in 2013 and in the multivariate model—in both analysed years.

People who used dental care least frequently were annuitants and pensioners. The individuals who resigned from dental care most often despite such a need were social welfare beneficiaries. It is not surprising that the situation of pensioners and annuitants as well as social welfare beneficiaries (in Poland these are usually unemployed persons) is less favourable since these two groups usually face the problem of exclusion (Manski et al. 2010; Patric et al. 2006). The univariate and multivariate logistic regression models showed that the group that resigned from dental care considerably less frequently was annuitants and pensioners when compared to waged and salaried individuals.

Considerable growth of the Gini coefficient of equivalised disposable income, (Eurostat 2017c) and a reduction of the percentage of self-reported unmet needs for dental examination (Eurostat 2017b) have been noticed in Poland in recent years. In our study, it was found that the use of dental services in the entire examined population did not change much in the analysed period (from 10.9 to 10.6%), yet the percentage of those who did not use dental care when in need dropped noticeably (from 7.6 to 4.2%). The frequency of use in the case of the highest group was more than twice as high as that in the case of the lowest group (as regards income, education, place of residence). These dependencies were confirmed also in the multivariate regression model. However, when both statistical methods were used, the differences in resigning from dental services despite such a need were clear only for income and place of residence. It can be assumed that people with a low SES are more willing to use dental care when in need, but they still use such care in general less often (for example, they resign from preventive services and examinations of the oral cavity).

The adopted methodology of a simultaneous comparison of the general use and non-use of dental services while in need depending on SES implies that the interventions concerning reduction of health inequalities ought to focus not only on the increasing availability of services to disadvantaged communities but also on health promotion and education (Artnik et al. 2008). Both community-based and individual intervention efforts should support behavioural change, which plays a crucial role in oral health. Extensive high quality observational studies, including prospective studies, are required in order to identify the most effective interventions in reducing health inequalities (Vandenbroucke et al. 2014).
